# Computational Biology in Cuba: An Opportunity to Promote Science in a Developing Country

**DOI:** 10.1371/journal.pcbi.0030227

**Published:** 2007-11-30

**Authors:** Tirso Pons, Luis A Montero, Juan P Febles

**Affiliations:** University of California San Diego, United States of America

## Science Policy and Bioinformatics in a Developing Country

Computational biology can be considered a supradisciplinary field of knowledge that merges biology, chemistry, physics, and computer science into a broad-based science that is important to furthering our understanding of the life sciences. Although a relatively new area of research, it is recognized as a crucial field for scientific advancement in developing countries. This Perspective introduces our vision of the role of computational biology in biomedical research and teaching in Cuba. Except where individuals are directly quoted, any opinions expressed herein should be considered those of the authors. (For author information, see Box 1.)

The challenge for Cuba was to initiate this new field of research and development without existing expertise. In addition, we had to face the same problems experienced elsewhere in this field—most life scientists were not familiar with the potential of information processing tools, and conversely, computer scientists were unfamiliar with problems facing the life sciences, often brought about by large amounts of new data. Cuba also faced unique problems. As a result of the United States trade embargo, Cuban scientists buy most of their research supplies from Europe. This adds delays and can triple costs, especially for equipment or spare parts that are made only in the US and must be purchased from a third party. In addition, curbs on travel between the US and Cuba isolate Cuban scientists from colleagues and conferences in the United States. Finally, Internet access is also affected by this policy, and Cuban scientists lack fast and efficient access to scientific information from overseas.

Despite these drawbacks, Cuban biotech products and activities from biomedical institutes within Cuba have been recognized by other authors [1–4]. Further offsetting these drawbacks, our country has a high educational level, and Cuban universities produce hundreds of new scientists every year. The challenge then is to implement higher education using both the coherence of existing disciplinary education and the means to break down disciplinary walls for students interested in computational biology and other multidisciplinary fields. Since Cuba has scant resources for significant investments in scientific instrumentation, a science such as computational biology that relies mostly on computers, network connectivity, and human resources offers an excellent opportunity. The current challenges facing computational biology research in Cuba are: (1) getting results of direct interest and impact to motivate funding, (2) fostering young leading scientists to sustain present and future research, and (3) using limited infrastructure in the most efficient and productive way.

Computational sciences in Cuba were first applied to physics and chemistry in the late 1960s at the University of Havana, with the use of quantum mechanics to model sugar cane derivative molecules [5]. Such an early application of this technology, which was entirely absent in the majority of the world, indicated a new approach to scientific development in a Latin American country. Another important effort began in the early 1970s at the National Center for Scientific Research in the field of computational neurosciences, mostly with Cuban-designed and Cuban-manufactured hardware [6].

Since then, after a heavy effort to build up human scientific potential, mostly with the influence and support of Eastern European countries, the problem of Cuba's limited computing potential was overcome and Cuban scientists gained valuable experience with microcomputers in the 1980s. Limitations to high-tech development during the 1970s originated from the obsolescence of devices built in the former Soviet bloc and a strong dependence on components from the same market, which hindered Cuba's national hardware production. Cuba was well-positioned to take advantage of the benefits of the microcomputer revolution of the 1980s. Applications of these new devices to the physical and biological sciences were frequent in that time and several computer programs were written or adapted to allow such applications [7,8]. During the economic crisis of the 1990s, these developments continued in the established groups and appeared in new teams at the Cuban Center for Genetic Engineering and Biotechnology (CIGB) [9].

A concerted attempt to meet the newest challenges of computational biology in Cuba began in 2000. An initiative arose from several senior scientists located at the above-mentioned Cuban research centers and several universities to promote new scientific disciplines and to establish interest among both the scientific community and national political leaders. (See Acknowledgments for a list of some of the researchers involved in this initiative.) This led to investment in computing resources in the main Cuban universities and research centers, providing Beowulf-like computer clusters for massive data processing, the inauguration of the National Bioinformatics Center (BIOINFO), and a joint effort to increase scientific human resources in a transdisciplinary way.

The opportunity to perform good science and to obtain important results that would directly impact Cuban society was a strong motivator. Today this implies identifying scientific problems with the greatest impact on human health, environmental concerns, sustainable production of food, and so on.

In addition to the investments made by different Cuban government agencies to create the above-mentioned significant computing facilities in some centers, a policy of establishing links to important foreign centers was promoted with scientific institutions in the United Kingdom, Belgium, The Netherlands, Italy, Germany, Brazil, and Spain. There are also scientific joint programs of co-tutorial doctorate education with Spain in this field. This cooperation encourages not only the exchange of scientific results of mutual benefit, but also graduate and Ph.D. education, mostly involving short-term student exchanges.

## Activities for Mutual Contacts and Cooperation

Based on this framework, Cuban researchers have organized and attended different seminars and international activities focused on computational biology in the last few years. These meetings, summarized in Table 1, have provided new collaborations and possibilities for developing national links and international projects contributing to computational biology research and education in Cuba.

**Table 1:**
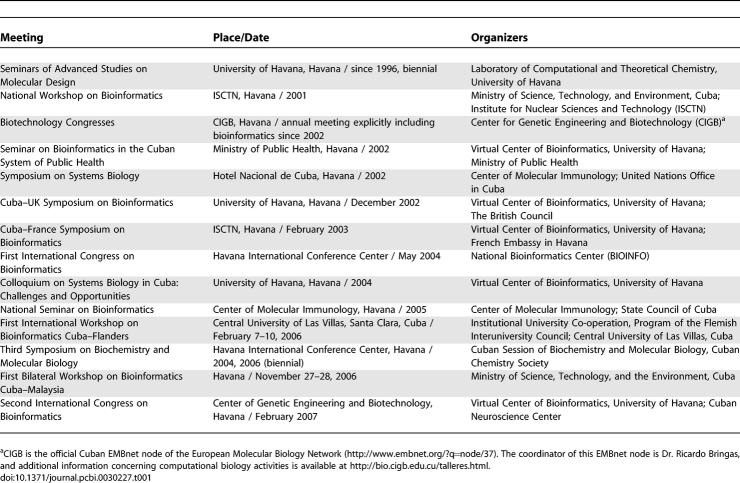
Meetings and Seminars Organized in Cuba on Computational Biology–Related Topics

One of them, the Seminars of Advanced Studies on Molecular Design, organized by the laboratory of one of the authors (LAM) at the University of Havana, has been held since 1986. In the beginning, these seminars were mostly dedicated to theoretical chemistry and molecular modeling. Since 2002, they have focused on life science applications. These meetings have provided a great opportunity to join top-level specialists and Ph.D. students for stimulating presentations and discussions. Among the oldest activities are the biotechnology congresses, sponsored by the CIGB, which began to include relevant computational biology topics in the late 1990s.

These international activities also led to the organization of national meetings, such as the Colloquium on Systems Biology in Cuba: Challenges and Opportunities, held at the University of Havana in 2004, and the National Seminar on Bioinformatics, held at the Center of Molecular Immunology in 2005. Binational meetings have also been held with specialists from the UK, France, Belgium, and Spain, promoting very fruitful discussions with Cuban science leaders and graduate students.

## Background and Topics in Computational Biology Research in Cuba

Almost all of the computational biology specialists leading research groups in Cuba hold a Ph.D. degree. Most performed their Ph.D. work only partly in Cuba, with the majority of the work done in Europe and to a lesser extent in Mexico and Brazil. The backgrounds of these scientists cover: sequence and 3-D structure analysis, functional residue prediction, comparative 3-D modelling, molecular dynamics simulations, molecular docking, mathematical model of T cell–mediated suppression, and studies of transcriptional regulation networks, as well as statistical and programming skills.

Interestingly, this initial group of computational biology specialists graduated in different fields (e.g., mathematics, physics, chemistry, and nuclear sciences), rather than in computational and biological disciplines as occurs in other places [10]. In recent years, an increased number of biologists and postdoctoral scientists from different fields have chosen to study computational biology because they need to apply new techniques to their own work or wish to improve their attractiveness to employers. Usually they do so by studying computational biology as part of their professional development, taking shorter, part-time, or distance-learning courses.

The diversity of graduating fields in computational biology explains the wide range of subjects and research topics currently under development in Cuba. These topics include: systems biology, the use of mathematical and statistical approaches to neurobiology, structure-based drug design, proteomics and genomics, mathematical biology, phylogenetic analysis of species unique to the region, protein–protein and protein–membrane interactions, computational genomics, protein design, and parallel processing of large datasets. A list of universities and research institutes developing comprehensive computational biology activities in the country is shown in Box 2.

## Pre- and Postgraduate Education

To develop a new multidisciplinary field of research in a country like Cuba, as elsewhere, requires the promotion and sponsorship of Master's and Ph.D. programs, either in Cuba or in foreign countries where the science is better developed.

Following this approach, four institutions—the Virtual Center of Bioinformatics, the Faculty of Biology at the University of Havana, BIOINFO, and the Central University of Las Villas—have created Master's and Ph.D. programs. The Virtual Center of Bioinformatics at the University of Havana also established conditions for national and international cooperative doctorate education. These programs have produced new scientific leaders, fast and relatively costless high-tech research results benefiting not only Cuban but also international cooperating institutions, and long-term cooperative projects.

Considering the role of computational biology in biomedical discovery, food production, agricultural research, and education, Cuban scientists view these programs as very important. Additionally, through this program, computational biology teaching at the undergraduate and graduate levels is receiving special attention. The first attempt at undergraduate education was an accelerated course on bioinformatics, organized by the CIGB, the Cuban Neuroscience Center, the University of Havana, and the Institute for Nuclear Sciences and Technology (ISCTN) for outstanding students in their final years of various majors.

At present, several career curricula from different Cuban universities include computational biology–related topics. For example, all undergraduate students of chemistry and biochemistry at the University of Havana take computer science, operating systems, elementary programming, and statistical courses as part of their basic training. In the case of the biochemistry major, as explained by Dr. Mayra Tejuca during the Third Symposium on Biochemistry and Molecular Biology held in Havana in October 2006, the new biochemistry study plan will allow students to choose other courses. These courses will include: Introduction to Bioinformatics, Computation, Advanced Statistics, Computational Study of Proteins, Computational Genomics, and Introduction to Systems Biology. Similarly, a computational engineering major at the University of Informatics Sciences (UCI) has an undergraduate curriculum that includes natural sciences. The purpose is to prepare engineers for interacting with basic field scientists to perform research and development on biological systems.

At the postgraduate level, an M.Sc. degree course organized by ISCTN in collaboration with the UCI is under development. Furthermore, three international cosponsored programs for a Ph.D. degree have been established. The first one, developed by the Universität Hasselt, Germany, and the University of Havana, Cuba, will provide a Ph.D. degree in Statistical Medicine and Bioinformatics. The second one, sponsored by the University of Valencia, Spain, and the Institute of Technology “José A. Echeverría,” Cuba, will provide a Ph.D. degree on Parallel and Distributed Processing on Computers and Development of GRID Applications for Bioinformatics. Finally, the Autonomous University of Madrid, Spain, and the University of Havana have recently initiated a cosponsored Ph.D. program in Bioinformatics. More recently, a Ph.D. program in Molecular Biosciences with new knowledge fields in Structural Biology, Bioinformatics, and Systems Biology has been initiated at the Faculty of Biology, University of Havana, sponsored by the CIGB and the Center of Molecular Immunology.

We believe that these meetings and teaching activities will provide more biologists, physicists, mathematicians, and chemists with both theoretical knowledge and practical skills in bioinformatics, while fewer and fewer jobs are available in these more traditional disciplines. With an increased number of computational biologists with M.Sc. and Ph.D. degrees, Cuba will hopefully host future meetings and contribute to scientific progress in computational biology.

## Conclusions

Computational biology represents a significant opportunity for a country like Cuba, where highly qualified human resources are available but funds to invest in equipment are scarce. In addition, the decentralized and noncompetitive cooperation between scientists and scientific institutions that has been budding recently creates a starting point for a promising national research field whose results will be useful not only scientifically, but for Cuban society as well. In the course of these developments, disciplinary barriers are being overcome, with scientists as educators implementing multidisciplinary programs in higher education. They have been reflected in the newest study plans for the Faculties of Biology, Chemistry, and Physics at the University of Havana that began in 2006. Thus, Cuba appears to be on the way to making a significant contribution to this new field of computational biology. 

##

**Box 1. Authors' Biographies**

**Tirso Pons Hernández** is the founder and group leader of the Laboratory of Computational Biology and Protein Design at the Center for Protein Studies, University of Havana. He obtained a B.Sc. degree in Nuclear Physics from the Institute of Nuclear Sciences, Havana, in 1991, and received a Ph.D. in Biology from the University of Havana in 2003. He is also adjunct professor at the University of Havana Biochemistry Department, where he teaches undergraduate and postgraduate courses in Bioinformatics. Dr. Pons was a visiting scientist at Dr. Alfonso Valencia's lab (CNB-CSIC) at the Autonomous University of Madrid, Spain, and at Dr. Gert Vriend's lab at the European Molecular Biology Laboratory, Heidelberg, Germany. He is a member of the Cuban Society of Biochemistry and Molecular Biology and has also received an invitation to become a member of the New York Academy of Sciences. Dr. Pons has received five Biomedical Science awards from the Cuban Academy of Sciences related to his scientific research.

**Luis Alberto Montero-Cabrera** has been Professor of Physical Chemistry at the University of Havana since 1983. He graduated from the University of Havana in 1969 and got his Ph.D. from the Technical University of Dresden, Germany, in 1980, before returning to Cuba and founding the Laboratory of Computational and Theoretical Chemistry at the University of Havana in 1986. Professor Montero teaches computer science for chemists in theoretical and physical chemistry, and has published abundantly in peer-reviewed journals in his fields of research, including science policy. He is chairman of the Scientific Council of the University of Havana 2006–2010 and has taught postgraduate courses in seven countries within Europe and in America, as well as authoring or adapting more than 15 computer programs in theoretical chemistry. Professor Montero has been awarded the honorary degrees “Carlos J. Finlay” for scientific research and “Frank País” for education. Both recognitions are among the highest Cuban national awards.

**Juan P. Febles Rodríguez** has worked as a Computing Professor at the Universidad de Matanzas since 1974. He obtained a B.Sc. degree in Mathematics from the Central University of Las Villas, Santa Clara, in 1974, and received a Ph.D. in Informatics from the Institute of Technology “José Antonio Echevarría,” Havana, in 1986. He took postgraduate courses at the Instituto Superior Politécnico de México (1979–1980) and at the Erevan Institute of Technology in Armenia (1984). In September 1996, he began working as Advisor at the Centro de Cibernética Aplicada a la Medicina, Instituto Superior de Ciencias Médicas, Havana, and was appointed Head of the Center in December 1997. He also teaches M.Sc. and Ph.D. courses in several universities in Cuba, Mexico, Brazil, and Guatemala. In the last five years, he has developed research projects in the areas of artificial intelligence, medical informatics, distance education, bioinformatics, and computer networks. He is currently Head of the National Scientific Program for Information Technology and a member of several national scientific groups.

**Box 2. Cuban Universities and Research Institutes Developing Computational Biology Research**

**•** Centro de Cibernética Aplicada a la Medicina

http://www.cecam.sld.cu/

**•** Center for Genetic Engineering and Biotechnology

http://bio.cigb.edu.cu/index.html

http://www.biocomp.cigb.edu.cu/

**•** Center of Molecular Immunology

http://www.cim.sld.cu/

**•** Centro de Química Farmacéutica

http://www.cqf.sld.cu/

**•** Cuban Neuroscience Center

http://www.cnic.edu.cu/

**•** National Bioinformatics Center

http://www.bioinfo.cu/

**•** Centro Nacional de Genética Médica

http://www.sld.cu/sitios/genetica/

**•** Centro Nacional de Sanidad Agropecuaria

http://www.censa.edu.cu/

**•** Finlay Institute

http://www.finlay.sld.cu/

**•** Instituto Superior de Tecnologías y Ciencias Aplicadas

http://instec.bioinfo.cu/

**•** Institute of Technology “José Antonio Echeverría”

http://www.cujae.edu.cu/

**•** Central University of Las Villas

*Faculty of Mathematics, Physics, and Computer Sciences*

http://www.mfc.uclv.edu.cu/

**•** University of Havana

*Faculty of Chemistry*

http://www.fq.uh.cu/investig/lqct/

*Faculty of Biology*

http://fbio.uh.cu/bioinfo/

http://fbio.uh.cu/cep/index.html

**•** Universidad de Oriente

http://www.uo.edu.cu/

## Abbreviations

BIOINFO: National Bioinformatics Center
CIGB: Center for Genetic Engineering and Biotechnology
ISCTN: Institute for Nuclear Sciences and Technology
UCI: University of Informatics Sciences
